# 

**Published:** 1902-09-15

**Authors:** 


### CONTENTS.

The X-ray as Applied to Dental Practice, *By L. E. Custer, D.D.S.* 433

Essential Oils; Their Application in Dentistry, *By David Stern, B.S., D.D.S.* 446

A Study in Bleaching Teeth, - *By N. S. Hoff, D.D.S.* 452

Clinics, - - - - - - - - - - - - 466

Editorial, - - - - - - - - - - - 472

Announcements, - - - - - - - - - 475

Bibliography, - - - - - - - - - - 476

Indents, - - - - - - - - - - - - 477

